# High-Fat Diet Induced Hedgehog Signaling Modifications during Chronic Kidney Damage

**DOI:** 10.1155/2020/8073926

**Published:** 2020-11-27

**Authors:** Rabia Mehmood, Nadeem Sheikh, Muhammad Babar Khawar, Muddasir Hassan Abbasi, Asima Tayyeb, Isbah Ashfaq, Maryam Mukhtar, Naz Fatima

**Affiliations:** ^1^Cell & Molecular Biology Lab, Department of Zoology, University of the Punjab, Q-A-Campus, Lahore 54590, Pakistan; ^2^State Key Laboratory of Stem Cell and Reproductive Biology, Institute of Zoology, Chinese Academy of Sciences, Beijing 100101, China; ^3^University of Chinese Academy of Sciences, Beijing 100049, China; ^4^Department of Zoology, University of Okara, Okara, Punjab, Pakistan; ^5^School of Biological Sciences (SBS), University of the Punjab, Q-A-Campus, Lahore 54590, Pakistan

## Abstract

Excessive consumption of dietary fats leads to the deposition of unnecessary metabolites and multiple organ damage. Lipids, important key regulators of Hedgehog signaling, are involved in triggering fibrotic chronic kidney disease. The present study encompasses the assessment of renal morphofunctional modifications and alteration of lipid metabolism influencing the changes in gene expression of hedgehog signaling pathway genes. Fifteen male *Rattus norvegicus* of 200 ± 25 grams weight were equally divided into three groups: control (standard rat chow), D-1 (unsaturated high-fat diet) and D-2 (saturated high-fat diet). Animals were provided with respective diets and were followed for 16 weeks. Both HFD-fed groups did not show overall body weight gain as compared to the control. While significant downregulation of hedgehog pathway genes was found in fatty diet groups. In comparison with the control group, *Shh*, *Gli1*, *Gli2*, and *Gli3* were downregulated after the consumption of both unsaturated and saturated fatty diets. *Ihh* and *Smo* exhibit a similar downregulation in the D-1 group, but an upregulation was detected in the D-2 group. D-2 group also had an increased serum urea concentration as compared to the control (*P* = 0.0023). Furthermore, renal histopathology revealed tubular necrosis, glomerular edema, glomerular shrinkage, and hypocellularity. Collagen deposition in both HFD groups marks the extent of fibrosis summary figure. Extravagant intake of dietary fats impaired normal kidney functioning and morphofunctionally anomalous kidney triggers on Hh signaling in adult rats. These anomalies can be linked to an escalated risk of chronic kidney disease in adults strongly recommending the reduced uptake of fatty diets to prevent impaired metabolism and renal lipotoxicity.

## 1. Introduction

The intrusion of extra dietary fats with intracellular lipids results in the deposition of detrimental lipids leading to inappropriate organelle functioning, cell damage, or even cell death [1]. Consumption of a high-fat diet (HFD) has a pivotal role in causing multiorgan damage including the liver, muscles, heart, pancreas, brain, and gut [2–5]. High lipid and sugar content in the Western Diet (WD) also results in the progression of metabolic anomalies. Consumption of HFD not only disturbs glucose tolerance but also causes loss of motor coordination and dyslipidemia [6]. Intake of high-fat diet leads to impaired lysosomal functioning and excessive deposition of phospholipids and cholesterol in renal tissues that ultimately lead to lipid metabolism alterations [7]. Surplus fatty acids supplemented with triglyceride deposition in the parenchyma of multiorgan tissues causes damage and chronic cellular dysfunction. HFD-induced nephropathy models have shown that increased lipogenesis and declined renal lipolysis contribute to intrarenal lipotoxicity. Lipid deposition in renal tissues prompts glomerular injuries and the formation of a tubulointerstitial lesion as in the case of type 2 diabetes and obesity [8–10]. The previous investigation on mice model have revealed that HFD consumption for sixteen weeks persuaded to lipid deposition in kidney and caused renal damage via albuminuria, interstitial fibrosis, and glomerulosclerosis [11].

The capability of grasping the signals responsible for adult health is constrained to a few signaling pathways including Wnt, Hippo, Notch, Hedge-hog, Jak/Stat, Tgf, and receptor-tyrosine kinase pathways [11]. Although limited in number, these signaling cascades can regulate a plethora of biological progressions accordingly. The developmental morphogen Hedgehog (Hh) has a potential role in metabolic control via downstream signaling [12]. Sonic hedgehog (Shh), Indian hedgehog (Ihh), and desert hedgehog (Dhh) are members of the Hh family that broadly cast a critical impact on biological events from embryogenesis to adulthood [13]. Hedgehog-Gli signal transduction contributes to the development of chronic kidney fibrosis. Hh ligands, Ihh, and Shh are found to be expressed in tubular epithelial cells. While the complete expression of Hh effectors, Gli1 and Gli2, is reported in perivascular fibroblasts and interstitial pericytes. Renal fibrosis models have depicted the escalated expression of Ihh accompanying Gli effector expression. In the course of fibrosis, interstitial cells (Hh responsive, Gli1 positive) experience proliferation along with the differentiation of Gli1 and Gli2 positive cells into myofibroblasts that are positive for *α*-smooth muscle actin. The onset of renal fibrosis may account for the activation of cell proliferation by Hh ligands pointing out the plausible role of this pathway in the progression of myofibroblast progenitors [14–16].

Lipids regulate Hh signaling pathway at multiple levels [17]. Hh binding to cholesterol via covalent linkage provides the basic clue for the lipid involvement in Hh signaling. These lipid modifications make Hh proteins stable in extracellular milieu [18, 19] and are obligatory for their distant transportation [20, 21]. Host lipid metabolism and mitochondrial dynamics are also found to be regulated via hedgehog signaling cascade in microorganisms [22]. Phospholipid metabolism markedly influences the regulation of the said pathway at Ptc/Smo level [22]. Hh signaling pathway has been found to have a check on lipid content in plasma [23]. In the course of renal injury, the embryonically conserved Hh signaling pathway is rebooted [14, 24–26].

So the excessive consumption of fats can possibly escalate the body's lipid content to abnormally high, disrupting the usual lipid metabolism. These changes can have the potential to trigger on Hh signaling pathway in the kidney of anomalous subjects. So, this investigation was aimed at exploring the changes in the expressions of fundamental genes that are involved in the Hedgehog signaling cascade and to assess the extent of morphofunctional change sin kidney due to the consumption of a high-fat diet in the Murine model.

## 2. Materials and Methods

### 2.1. Development of Animal Model

Adult male *Rattus norvegicus* of 13 ± 1 weeks of age and 200 ± 25 g weight were used in this study. The present investigation was conducted following the institutional guidelines approved by the Local Ethical and Review Committee of the Department of Zoology, University of the Punjab, Lahore, Pakistan (Ref.D/621/U.Z). Five animals were housed per cage preceding the experiment provided with controlled environmental conditions reported previously [27]. Rats were randomly assigned to three groups: control, D-1, and D-2 (n = 5). The control group was fed on standard rat chow throughout the experiment. The experimental group “D-1” was served with high-fat diet 1 (HFD1) (Table 1). The other experimental group “D-2” was provided with high-fat diet 2 (HFD2) (Table 1). All animals had *ad libitum* access to their respective diets and drinking water for sixteen (16) weeks. The weight of animals was checked twice-weekly throughout the experimentation period. After 16 weeks of high-fat diet supplementation, an equal ratio of ketamine and pyrogen-free water was administered intraperitoneally to anesthetize animals. Blood was drawn through direct cardiac puncture as described by Abbasi et al. [28]. After euthanizing, kidneys were removed in two portions: one part was formalin (10%) fixed for histochemical examination, and the other part was snap-frozen for gene expression analysis at -80°C summary figure.

### 2.2. Quantitative Real-Time PCR

Total RNA was extracted from experimental and control groups using TRIzol® reagent (Invitrogen USA, 10296028) following the manufacturer's guidelines. Extracted RNA was passed through DNase treatment (QIAGEN, RNase free-DNase set) as per the manufacturer's instructions. Total RNA was photometrically quantified (UV-Vis SpectrophotometerNanoDrop™2000, Thermo Scientific, USA). RevertAid First Strand cDNA Synthesis Kit (Thermo Scientific, K1622) was used to synthesize cDNA. Real-time PCR was performed using qPCR reaction mixture (see supplementary data for details) on the PikoReal™ Real-Time PCR System (Thermo Scientific) and the enlisted primers (Table 2). Results were analyzed by the PikoReal software (2.2; Thermo Scientific). Briefly, the qPCR samples were prepared using Maxima SYBR Green/ROX qPCR Master Mix (K0221, Thermo Scientific) (see supplementary materials (available here)). PCR cycles were performed at 94°C for 30 s, 55-62°C for 30 s, 72°C for 30 s, 72°C for 10 min, and then held at 4°C after the optimization of best reaction conditions using a temperature gradient (see supplementary materials (available here)). To balance the potential irregularities in RNA concentration, all values were normalized to GAPDH (Table 2). A negative control (RT^−^) was concordantly added to each qPCR reaction to control the affectivity of the procedure. Fold changes in expression of the said genes were calculated by 2^*ΔΔ*Ct^ method.

### 2.3. Measurement of Serum Urea and Creatinine

Under sterile conditions, hemolysis free serum samples were separated as reported previously [29] and employed to investigate renal functioning tests. Ready to use kits were used for serum urea and creatinine estimation as per the manufacturer's guidelines. Ammonia, one of the hydrolysis products of urea, reacts with alkaline hypochlorite and sodium salicylate in the presence of sodium nitroprusside to yield a green chromophore. The color intensity of which corresponds to sample urea concentration. While serum creatinine estimation is based on the principle that creatinine reacts with picrate ions to form a red-colored complex in an alkaline medium. Increasing absorbance that is proportional to the rate of formation of the complex corresponds to the amount of creatinine in the sample.

### 2.4. Histochemical Analysis

Standard methods were used to process formalin-fixed renal tissues for Hematoxylin and Eosin (H&E) and Masson's Trichrome staining from Sigma-Aldrich following manufacturer guidelines.

### 2.5. Statistical Analysis

Data sets were evaluated employing the Prism GraphPad 5 software (San Diego, CA). Statistically significant differences among groups were identified by using one way ANOVA (analysis of variance) and Kruskal-Wallis test followed by *post hoc* Tukey's test and post-Dunn's test respectively. *P* values that were less than 0.05 were accepted to be significant.

## 3. Results

### 3.1. Bodyweight

**Figure 1:**
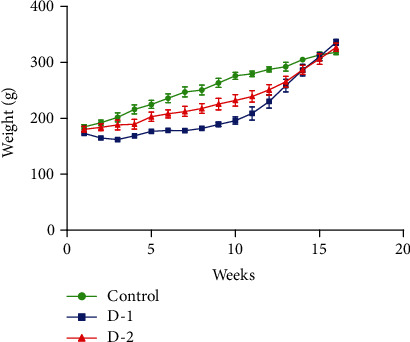
Weight gain in D-1 and D-2 groups as compared to control throughout experimental span. (D-1 = Murine model fed on high-fat diet 1; D-2 = Murine model fed on high-fat diet 2).

There was no overall difference noticed in the bodyweight of organisms among groups before and/or after following the experimentation (Figure 1).

### 3.2. Gene Expression of *Shh*, *Ihh*, and *Smo*

**Figure 2:**
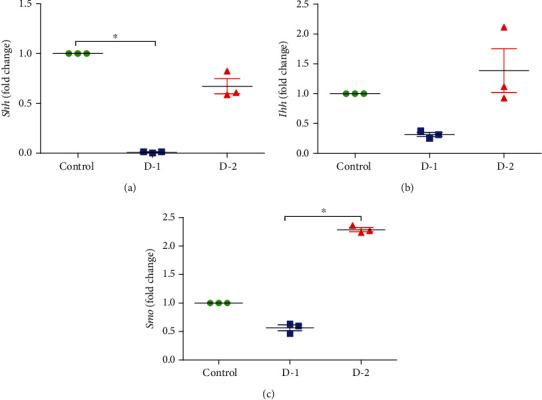
Quantitative mRNA expression of (a) *Shh*, (b) *Ihh*, and (c) *Smo* in D-1 and D-2 as compared to control. (D-1 = Murine model fed on high-fat diet 1; D-2 = Murine model fed on high-fat diet 2). qRT-PCR statistics showed fold changes in the level of mRNA expression after normalization with housekeeping gene GAPDH. Statistically significant variations are marked by the asterisk (^∗^*P* < 0.05, ^∗∗^*P* < 0.01, ^∗∗∗^*P* < 0.001; Mean ± S.E.M.; *n* = 5).

To assess any change in the expression of the hedgehog signaling pathway, we performed quantitative real-time PCR on a few important genes of the hedgehog signaling pathway. Statistically marked variations were observed in *Shh* expression of the high-fat diet-fed animal model compared to the control. Additionally, *Shh* expression was decreased in D-1 as compared to control (*P* = 0.0241). The expression was escalated in D-2 as compared to D-1 compared. D-1 showed a decreased expression of *Ihh*, while the expression of *Ihh* was higher in D-2. A difference in *Ihh* expression between the two study groups was revealed by post-Dunns's test. *Smo* expression was also changed in both experimental groups compared to control. *Smo* was downregulated in D-1, while an increase of 1.287 folds was observed in the D-2 experimental group (Figure 2).

### 3.3. Gene Expression of *Gli1*, *Gli2*, and *Gli3*

**Figure 3:**
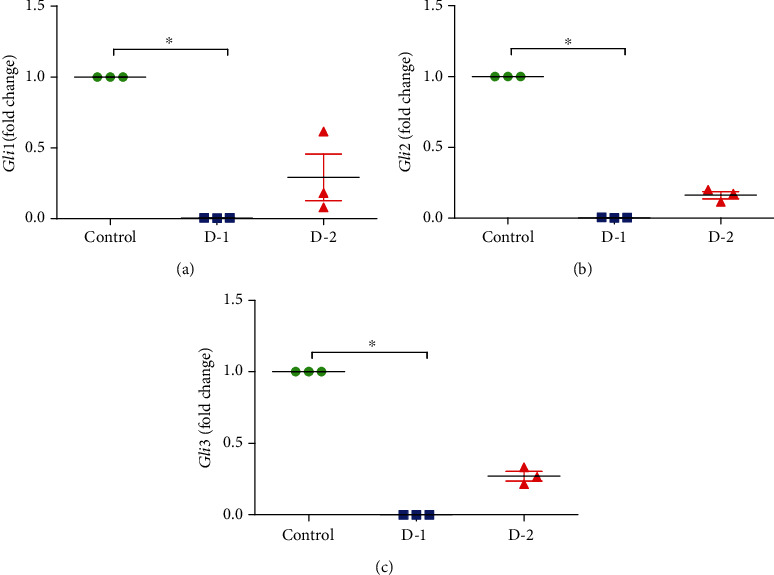
Quantitative mRNA expression of (a) *Gli1*, (b) *Gli2*, and (c) *Gli3* in D-1 and D-2 as compared to control. (D-1 = Murine model fed on high-fat diet 1; D-2 = Murine model fed on high-fat diet 2). qRT-PCR statistics showed fold changes in the level of mRNA expression after normalization with housekeeping gene GAPDH. Statistically significant variations are marked by the asterisk (^∗^*P* < 0.05, ^∗∗^*P* < 0.01, ^∗∗∗^*P* < 0.001; Mean ± S.E.M.; *n* = 5).

A parallel statistically significant decrease in *Gli1* expression was found in HFD1-fed experimental group. In line with it, *Gli2* and *Gli3* also showed a similar pattern of expression among all the experimental groups. Chronic consumption of HFD1 led to a significantly decreased expression of *Gli2* and *Gli3* as compared to control (*P* = 0.024001) (Figure 3).

### 3.4. Urea and Creatinine Level

**Figure 4:**
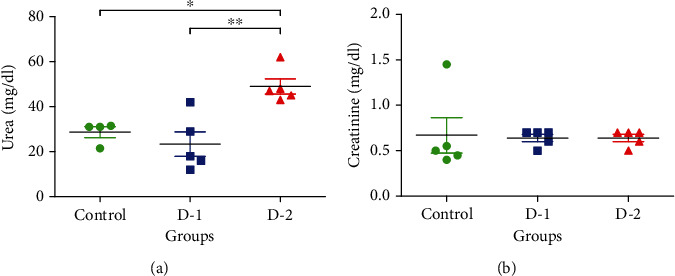
Renal functioning tests. The level of (a) urea and (b) creatinine in treated groups D-1 and D-2 against control. (D-1 = Murine model fed on high-fat diet 1; D-2 = Murine model fed on high-fat diet 2). Statistically significant variations are marked by the asterisk (^∗^*P* < 0.05, ^∗∗^*P* < 0.01, ^∗∗∗^*P* < 0.001; Mean ± S.E.M.; *n* = 5).

Next, to find out whether HFD has affected the function of the kidney, we checked the level of serum urea and creatinine. We found a 30% drop and 45% uprising of serum urea level in D-1 and D-2 experimental groups, compared to the control. Urea concentration in D-2 varied significantly from control and D-1 group as well. However, we did not find any significant change in creatinine levels in both experimental groups as a result of HFD supplementation (Figure 4).

### 3.5. Histochemical Analysis

**Figure 5:**
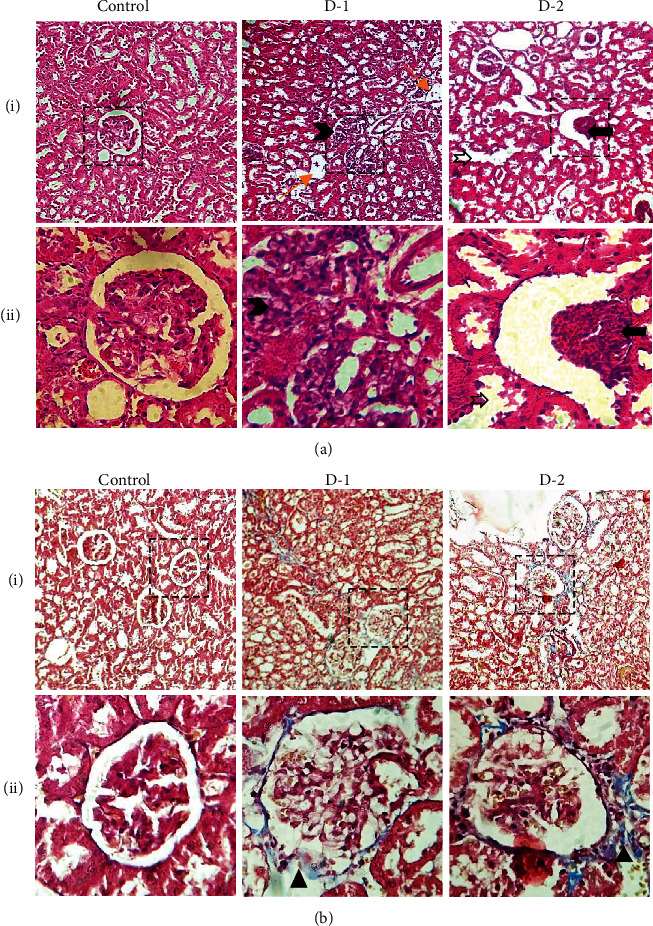
Microphotographs of (a) hematoxylin and eosin-stained renal sections of control and treated groups D-1 and D-2. Glomerular edema (➱) and tubular necrosis (yellow rightwards arrow) can be noted in the D-1 renal section, while hypocellularity (❱) and shrinkage of glomeruli (⬅) marked the histological changes in D-2 and (b) of Masson's trichrome stained renal sections of control and treated groups D-1 and D-2. Blue color along the glomerular capsule ▲) is an indication of the collagen deposition in both study groups. (D-1 = Murine model fed on high-fat diet 1; D-2 = Murine model fed on high-fat diet 2). (Magnification; i: 100x; ii: 400x).

To further confirm the effects of a high-fat diet on kidneys, we carried out a histological examination on H&E-stained kidney sections. Histological examination of control kidney sections showed a comprehensive microarchitecture comprised of nephrons and collecting ducts; thus, uriniferous tubules showed up intact functional units and no damage to the kidney. In the experimental group, D-1 infiltration of leukocytes, dilation of Bowman's capsule, and degeneration of tubular cells with pyknotic nuclei were present. Glomerular edema and tubular necrosis were also evident in renal tissues of animals chronically fed with a high-fat diet. Similarly, HFD2 consumption posed extensive cortical damage, shrinkage, and hypocellularity of the impaired glomeruli in the D-2 group (Figure 5(a)). Next, to evaluate the degree of fibrosis, we carried out Masson's trichrome staining and found a remarkable collagen deposition along with the capsular region in both experimental groups compared to the control (Figure 5(b)).

## 4. Discussion

**Figure 6:**
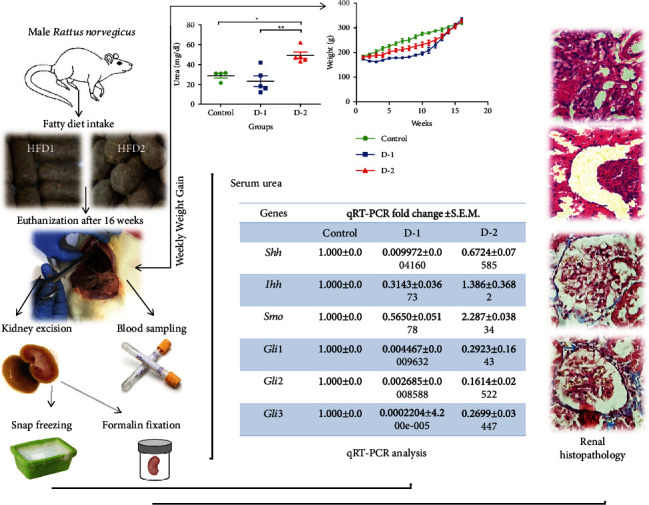
HFD induces remarkable variations in hedgehog pathway genes; especially, *Shh*, *Gli1*, *Gli2*, *Gli3*, *Ihh*, and *Smo* were downregulated. Serum urea concentrations were elevated and tubular necrosis, glomerular edema, glomerular shrinkage, and hypocellularity were induced in kidneys. Fibrosis was also confirmed by significant collagen deposition in both HFD groups. Too much dietary intake of fats impaired normal kidney functioning and fibrotic kidney triggers on Hh signaling in adult rats suggesting chronic kidney disease.

In the present study, we investigated the morphofunctional changes associated with the kidney in response to chronic exposure to unsaturated and saturated fatty diets. Besides the potential changes in the expression of key genes, i.e., *Shh*, *Ihh*, *Smo*, *Gli1*, *Gli2*, and *Gli3* in the Hedgehog signaling pathway were explored. Furthermore, fluctuations of serum levels of urea and creatinine along with renal histopathology are correlated with the said modifications (Figure 6).

After 16 weeks of HFD, both HD groups were presented with no difference in overall weight gain. This information corroborates with the former investigations regarding HFD in animal models, where HD groups showed up the lesser intake of respective diets, and an increase of body weight was not observed. Rather, statistically escalated adiposity was demonstrated [30, 31]. Genetic, behavioral, physiological, and several other mechanisms work together to regulate body weight. An imbalance between food intake and energy expenditure results in health penalties [32].

Amendment in the stability of lipogenesis and lipolysis followed by lipid deposition in the kidney has been experimentally proven in HFD-fed animal models [11]. Besides, tissue impairment is a determinant of activated developmental pathways, and several studies have reported the association of fibrosis with activation and enhanced expression of Hh signaling. The incited Hh pathway can pursue the proliferation and transformation of myofibroblasts [33].

Sonic hedgehog (*Shh*) is a renowned hedgehog ligand that is concomitant with kidney development and repair of renal tissues [26]. While acting as a paracrine signal, Shh endorses mesenchymal proliferation and checks the differentiation time of smooth muscle progenitor cells [34]. The tubular epithelium is the fundamental target during renal damage. Interstitial fibroblasts are the intrinsic target of *in vivo* Shh signaling whereas *in vitro* growth with Shh encouraged fibroblast proliferation in normal rats [35]. Indian hedgehog (*Ihh*) is expressed in the adult kidney [36] While both *Shh* and *Ihh* are expressed in tubular epithelia [14].

Significant downregulation of the majority of genes involved in Hh signaling was observed. *Shh* was significantly downregulated in both experimental groups as compared to control. A similar trend of decreased expression was found for *Ihh* and *Smo* in the D-1 study group. While contrary to control and D-1, D-2 demonstrated an upregulation of *Smo*. After kidney injury, intercellular communication is fetched out by Shh through interstitial fibroblast targeting. Former studies have concluded that active Shh/Gli signaling endorse the assembly of the extracellular matrix, activation of fibroblasts, and cause interstitial renal fibrosis [25]. Obstinately, literature studies also reveal upregulated *Ihh* level in the course of renal fibrosis that leads to the downstream activation of Gli expression [14].

Transcriptional Hh activator Gli family comprised of three distinct proteins Gli1, Gli2, and Gli3 in vertebrates [37]. It has been reported that *Gli2* is one of the chief activators of Hh signaling, while *Gli3* is the repressing one. The role of downstream signal amplification is most likely to be played by *Gli1* [37–41]. In current exploration, a marked significant decrease in expression of *Gli1*, *Gli2*, and *Gli3* was found among both study groups when compared with the expression of these changes in control. In humans, the severity of interstitial fibrosis marked the significance level of expression of *Gli* and *Gli 2*, which was found to be significantly upregulated in fibrosis (<80%) as compared to inferior grade, i.e., <20% interstitial fibrosis [42].

A strong link has also been shown between renal fibrosis and dysregulated Shh signaling previously [26]. Activation of Hh pathway found its probable importance in the regeneration of damaged organs but excessive stimulation can also take part in developing fibrosis. Reduced Hh signaling holds potential in building up therapeutic stratagem to ameliorate the onset of chronic kidney disease (CKD) [33]. Genetic ablation of key cells and/or pharmacologic interventions can inhibit Shh/Gli signaling that can stop fibrosis after injury [44, 45].

Serum urea level was significantly escalated in the D-2 group as compared to the control and D-1 study group. Histopathological investigations lead to explore glomerular edema and tubular necrosis in D-1, whereas hypocellularity and shrinkage of glomeruli in D-2 highlighted the drawbacks of consumption of fatty food for a longer period. Collagen deposition was also found in both D-1 and D-2 that pointed out the kidney fibrosis. Excessive inflammation and epithelial damage refer to fibrosis that ultimately results in the activation of myofibroblasts. Kidney fibrosis usually holds a histological appearance in depicting the functional deterioration of the kidney [33]. From surplus fats, triglycerides carry storage function, while nonesterified fatty acid (NEFA) component mainly owes toxicity. Previous investigation has shown that NEFA magnified the chronic tubule damage and inflammation that resulted in nephropathy [9].

Excessive intake of saturated and/or unsaturated fatty diet over a long period triggers the embryonically conserved Hh pathway in adult impaired kidney. Initially, this activation helps to combat fibrosis but prolonged activation can potentially worsen the scenario. Besides, morphofunctional deteriorations of kidney strongly condemn fatty diet consumption that is not yet all taken up seriously in most of the geographical realms especially in the Indo-Pak subcontinent. There must be enhanced public awareness, improved food quality, obligatory food labels, prevention of the young generation from destructive food marketing, and accessibility of healthy food for everyone. A well-balanced diet plan and stupendously active physical routine can ameliorate overall health and body weight.

## Figures and Tables

**Table 1:** Composition of commercially available laboratory chow, HFD1, and HFD2 confirmed through the analysis performed at PCSIR (Pakistan Council of Scientific and Industrial Research) complex laboratories, Lahore, Pakistan.

Ingredients	% composition		
	Standard rat chow	HFD1	HFD2
Fat	7.17	33.37^∗^	33.37^#^
Protein	17.2	6.88	6.88
Carbohydrate	51.48	47.51	47.51
Fiber	5.49	2.20	2.20
Ash	9.31	5.19	5.19
Moisture	8.9	4.85	4.85

^∗^Highly unsaturated fats; ^#^highly saturated fats.

**Table 2:** List and sequences of RT-PCR sequences.

Primers	Forward 5′⟶3′	Reverse 3′⟶5′
GAPDH	GAAACCTGCCAAGTATGA	GCTGTAGCCGTATTCATT
Shh	CAATTACAACCCCGACATCA	AGTCACTCGAAGCTTCACTCC
Ihh	TCAGCGATGTGCTCATTTTC	CCTCGTGAGAGGAGCATAGG
Smo	AGAAGGCCTTGGCAATCA	GAAGCCCATTCCTGATTGTG
Gli1	TGGAAGGGGACATGTCTAGC	GCTCACTGTTGATGTGGTGC
Gli2	CAGTGGCAGTTGGTCTCGT	ATAAGCGGAGCAAGGTCAAG
Gli3	CCCTCTCTCCCTTATCGTG	AAGGCAAGTCTGGATACGTT

## Data Availability

No additional data has been utilized in the present study.

## Abbreviations

CKD: Chronic kidney disease
Dhh: Desert hedgehog
H&E: Hematoxylin and eosin
HFD: High fat diet
Hh: Hedgehog
Ihh: Indian hedgehog
NEFA: Nonesterified fatty acid
Shh: Sonic hedgehog
WD: Western Diet.

## Supplementary Materials

Supplementary Materials Table 1: Composition of final qPCR reaction mixture (12*μ*l). Table 2: qPCR Cycling Conditions.
