# Supplementary material for: High-Fat Diet Induced Hedgehog Signaling Modifications during Chronic Kidney Damage
Source: Biomed Res Int. 2020 Nov 27;2020:8073926. doi: 10.1155/2020/8073926 (PMC7718043; doi:10.1155/2020/8073926)
Supplement: Supplementary Materials — Table 1: Composition of final qPCR reaction mixture (12μl). Table 2: qPCR Cycling Conditions. [file 8073926.f1.docx]

**Supplementary Data**

**Composition of final qPCR reaction mixture (12µl)**

| **Component** | **Volume (µl)** |
| --- | --- |
| SYBR Green | 6 |
| Forward Primer | 1 |
| Reverse Primer | 1 |
| cDNA | 1 |
| DEPC Water | 3 |

**qPCR Cycling Conditions**

| **Temperature °C** | **Time** | |
| --- | --- | --- |
| 94 | 5 min | |
| 94 | 30 s | 40 Cycles |
| 55-62 | 30 s |  |
| 72 | 30 s |  |
| 72 | 10 min | |
| 4 | Hold | |
